# Valorization of Fish by-Products: Purification of Bioactive Peptides from Codfish Blood and Sardine Cooking Wastewaters by Membrane Processing

**DOI:** 10.3390/membranes10030044

**Published:** 2020-03-13

**Authors:** Soudabeh Ghalamara, Sara Silva, Carla Brazinha, Manuela Pintado

**Affiliations:** 1 Centro de Biotecnologia e Química Fina—Laboratório Associado, Escola Superior de Biotecnologia, Universidade Católica Portuguesa/Porto, Rua de Diogo Botelho, 1327, 4169-005 Porto, Portugalsnsilva@porto.ucp.pt (S.S.); mpintado@porto.ucp.pt (M.P.); 2 LAQV/Requimte, Faculdade de Ciências e Tecnologia, Universidade Nova de Lisboa, Campus de Caparica, 2829-516 Caparica, Portugal

**Keywords:** fish by-products, bioactive peptides, membrane processing, ultrafiltration, biorefinery

## Abstract

Codfish blood and sardine cooking wastewaters were processed using membrane ultrafiltration that allowed for the preparation of bioactive peptides enriched fractions. The raw materials and corresponding permeates were characterized chemically and in terms of biological properties. The fractionation process was evaluated by analyzing the selective permeation of small peptides (<1 kDa) from larger compounds when using membranes with different molecular weight cut-offs (MWCOs) combined with different materials (MW, PW, and UP010 for codfish blood) and when operated at different transmembrane pressures (with GH for sardine cooking wastewaters). A rejection of the protein/peptides >10 kDa was achieved for both raw materials with the studied membranes. Also, low values of rejection of peptides <1 kDa were accomplished, namely 2% with UP010 from codfish blood and 23% when operated at minimum pressure (1.0 bar) with GH from sardine wastewaters. The peptide fractions from codfish blood with MW and UP010 exhibited the highest ABTS^+^ and ORAC values. Peptide fractions from sardine wastewaters with GH demonstrated no improvement in antioxidant activity compared to sardine wastewaters. The antimicrobial results showed that the peptide fractions from codfish blood with UP010 and from sardine with GH at 1.0 bar were capable of inhibiting *Escherichia coli* growth.

## 1. Introduction

For many countries around the world, the fishing industry is a pillar of their economy. According to the Food and Agriculture Organization (FAO), the world fish production in 2016 was estimated at 174.1 million tons (mt) for both capture and aquaculture [1]. A billion people are directly or indirectly dependent on trade and fish production [2]. More than 60% (weight) of the processed fish is estimated to be by-products (such as heads, skins, bones, fins, trimmings, viscera, blood, and roe) [3]. These by-products are mostly discarded without recovery intention and have a considerable negative ecological impact [4,5]. Therefore, the valorization of fish by-products could have a positive impact on the economic viability of the fishing and aquaculture industry, namely through the production of value-added ingredients attained using effective technological solutions. Fish by-products could be a source of value-added compounds like proteins, peptides, and amino acids [6]. Numerous studies have shown that the crude protein content of fish by-products ranged from 8 to 35% [7]. Fish processing by-products and underused catch, containing a large quantity of protein, are generally converted into low-value products such as animal feed, fishmeal, and fertilizers [1].

Biopeptides can be naturally found in fish by-products. These biopeptides (usually contain no more than 20 amino acids) are capable of modulating physiological processes and, therefore, may have a role in the prevention and control of diseases. Oxidative stress is an essential factor that contributes to the development and progression of chronic non-communicable diseases (NCDs). Peptides from fish processing by-products, which exhibits antioxidant activity (such as VKAGFAWTANQQLS (1519 Da)), have been purified from tuna backbone [8]. The ORAC activity reported for peptides derived from fish processing by-products has been reported to vary from 5.47 to 19.74 μmol TE peptide/μmol peptide. The antioxidant activity of biopeptides has been associated to the presence of specific amino acids, such as histidine residues. The peptide purified from the Alaska pollack (*Theragra chalcogramma*) frame protein contained a histidine residue (Leu-Pro-His-Ser-Gly-Tyr (672 Da)), exhibited potent antioxidant activity [9]. This can be attributed to the chelating and lipid radical-trapping capacity of the imidazole ring. Moreover, the antioxidant activity of histidine-containing peptides was greater than that of histidine itself, partly due to an improvement in peptide and fatty acid hydrophobicity [10,11].

High blood pressure plays an important role in the development of an array of diseases and is one of the major risk factors for congestive heart failure, myocardial infarction, stroke, arteriosclerosis, and renal failure [12,13]. The angiotensin I-converting enzyme (ACE) is used physiologically to transform angiotensin I to angiotensin II, an active vasoconstrictor peptide that controls blood pressure and inactivates the vasodilator bradykinin [14]. Given the rise in the prevalence of cardiovascular diseases, these have led to an increase in the search for new ingredients that could contribute to the reduction of hypertension. Peptides are potential active ingredients that could be used for this purpose. Lee et al. [15] identified two ACE inhibitory peptides in the skin of skate (*Raja Kenojei*) with IC50 values of 95, and 148 mM and the peptides act against ACE as non-competitive inhibitors. This study suggested that novel ACE inhibitory peptides obtained from skate skin protein could be useful as anti-hypertension compounds in functional foods. In vitro, fish-scale collagen peptides reduced the expression of pro-inflammatory cytokines [16]. Several peptides with ACE inhibitory activity have been identified from bluefin tuna [17], leatherjacket [18], Atlantic salmon [19,20], boarfish [21], rockfish [22], skate [23], and *Pangasius* catfish [24].

Antimicrobial peptides are biomolecules used by animals and plants to protect against bacteria [25]. They are, typically, positive-charged short-chain peptides comprised of 12–45 amino acid residues. Several antimicrobial peptides have been derived from marine fishes, such as winter flounder, *Pleuronectes americanus*, *American plaice*, *Hippoglossoides platessoides*, *Atlantic halibut*, and *Hippoglossus hippoglossus* [26,27]. Several biopeptides with specific molecular weights from fish by-products with antioxidant, antimicrobial, and ACE inhibitory activities are represented in Table 1.

Considering the high amount of proteins and potentially bioactive peptides present in fish by-products, along with the increasing global demand for high quality protein/peptides, there is a growing interest in the purification of these compounds. Pressure driven membrane technology operated under standard conditions (the concentration mode of operation under controlled transmembrane pressure conditions) is suitable for the purification of protein/peptides, as it is a sustainable technology needing neither solvents nor absorbents. Additionally, it is an industrially established, non-expensive technology, when compared to electrically driven membrane technology and to the different chromatography techniques. Considering this size, ultrafiltration (UF) has a range of molecular weight cut-offs (MWCOs) appropriate to purify most of proteins and peptides. UF processes are reported in the literature, specifically for this application. Chabeaud et al. [35] used a UF membrane in a UF/NF Microlab40 pilot plant (VMA Industrie, France) to improve the bioactivity of a pollack protein hydrolysate by producing a fraction of the hydrolysate enriched in peptides with molecular weights (MWs) < 7 kDa. That was achieved by selecting a membrane with a MWCO of 4 kDa with modified polyethersulfone (PCI, ref. ESP04 (France)), which favored the permeation of peptides within the MW target range and rejected peptides with much higher MWs.

Afonso et al. [36] evaluated the technical and economic viability of protein recovery, from Mackerel fish meal effluents, using an UF ceramic membrane (Carbosep M2, monotubular, 15 kDa MWCO, with an active layer of ZrO_2_–TiO_2_ on carbon support) and a nanofiltration (NF) membrane (Kerasep NanoN01A, tubular, 1 kDa MWCO, with an active layer of ZrO_2_ or TiO_2_ on ceramic support, Rhodia–Orelis). Mackerel processing effluents were pretreated with microfiltration (MF) cartridges battery (Omnifilters of decreasing pore sizes: 80, 20, and 5 µm) followed by UF or NF. The selected treatment for fishmeal effluents, pretreatment with MF followed by UF, allowed a 69% recovery of proteins and a significant reduction in environmental burdens. The economic evaluation of protein recovery, by UF membrane, from fish meal effluents was carried out for the production of 544 ton/y of fish meal, yielding a net present value of 160 × 10^3^ US$, 17% interest rate of return and eight-year payback time. Therefore, the use of UF membranes in fishmeal effluents is, theoretically, economically feasible to recover protein and reduce pollution.

Picot et al. [37] studied the impact of a UF membrane (polyethersulfone, 4 kDa MWCO, reference ESP04) and an NF membrane (polyamide/polyethersulfone, 300 Da, 60% retention in CaCl_2_, reference AFC40) on the stability of biological activities of Pacific cod skin gelatin. Successive fractionation with UF and NF membranes permitted the enrichment of peptides, with an MWCO appropriate to the peptide composition, providing an effective way of concentrating gene-related peptide (CGRP) like peptides and peptides enriched in selected amino acids.

Pezeshk et al. [38] used UF membranes (3, 10, and 30 kDa MWCO; with regenerated cellulose; from Fisher Scientific, Oakville, ON, Canada) to evaluate antioxidant and antimicrobial activities of yellowfin tuna (*Thunnus albacores*) viscera peptides. The results showed that the lowest molecular weight fraction (< 3 kDa) had significantly higher levels of bacteria inhibition against Gram-positive (*Listeria and Staphylococcus*) and Gram-negative (*E. coli* and *Pseudomonas*) pathogens and fish spoilage-associated microorganisms as well as scavenging activity against DPPH, ABTS^+^ radical, and ferric reduction.

The case-study of this work is focused on small biopeptides from codfish and sardine by-products, respectively, blood and wastewaters. The chosen raw materials are among the most processed species in Portugal, which are disposed of with high and expensive environmental impact. The aim of this work was to demonstrate the feasibility of using (pressure-driven) membrane technologies to purify small biopeptides from codfish blood and sardine cooking wastewaters (target compounds) and to optimize this process. The target compounds aimed to be preferentially permeated through UF membranes, removed from the large compound contaminants. Additionally, the permeate fraction (enriched in the target compounds) should have enhanced biological properties when comparing to the raw materials. To the best of our knowledge, the purification of these raw materials by UF, complemented with the characterization of the chemical composition and biological properties of the raw materials and corresponding permeates has not still been reported.

For a better understanding of the mass transport phenomena during fractionation by membrane processing, UF membranes used exhibited different membrane MWCO and well comprised of different membrane materials and operated under different transmembrane pressures. The fish by-products and the corresponding fractions (retentate and permeate obtained from membrane processing) were chemically characterized in terms of their content in protein/peptides by Kjeldahl and the fast protein liquid chromatography (FPLC) methods. The selection of the MWCO of the membranes was made after the characterization of the raw materials by FPLC. The fish by-products and permeate fractions were characterized in terms of biological properties, specifically their antioxidant, antimicrobial, and antihypertensive activities. The optimization of the membrane processing involved the selection of the most appropriate UF membrane and the selection of the best mode of operation in an attempt to produce active ingredients that may be exploited by the food, pharmaceutical, or cosmetic industries.

## 2. Materials and Methods

### 2.1. Materials

Codfish blood and sardine cooking wastewaters were kindly provided by Pascoal S.A. (Aveiro, Portugal) and A Poveira S.A. (Porto, Portugal), respectively. After collection, they were immediately transported and stored at -20 °C until processing. Before membrane processing, the raw materials were pre-treated: The codfish blood was deodorized at 30 mbar and 25 °C with a rotary evaporator (Büchi R-210, Flawil, Switzerland). The deodorized codfish blood and the sardine cooking wastewaters were centrifuged (Sigma 4K15, Sartorius, Epsom, UK) for 10 min, at 20 °C and 8000 rpm to remove solid components. Before further use, the samples were frozen at −20 °C for storage.

The membranes selected for this work (which selection is explained in Section 3.1) are described in Table 2.

### 2.2. Experimental Procedure

**Figure 1:**
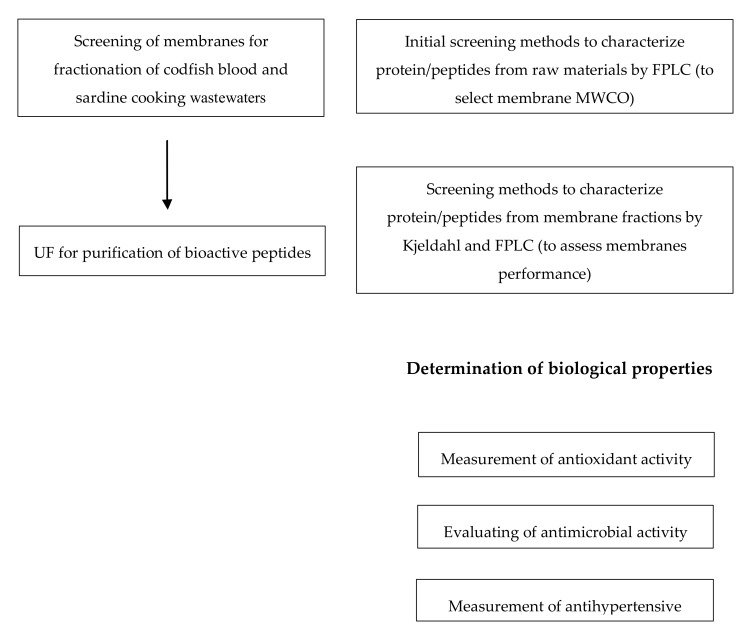
Flow diagram summarizing the experimental studies performed in this work.

The scheme of the different experimental studies performed in this work is presented in Figure 1. In order to select the fractions enriched in biopeptides from the raw materials under study with maximized biological properties, the quality of the different permeates produced by ultrafiltration will be assessed by the quantification of the peptides/proteins present, also discriminated in their sizes (by FPLC), as well as by the determination of different types of biological activities (specifically antioxidant, antimicrobial and antihypertensive activities).

#### Membrane Processing

**Figure 2:**
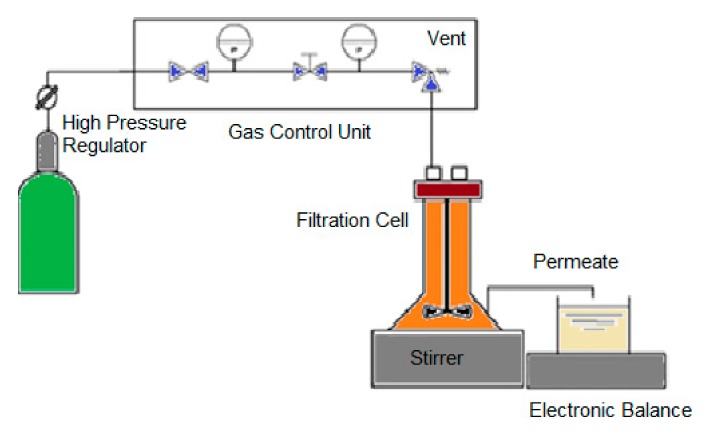
Scheme of the membrane processing used in this work to purify the biopeptides from codfish blood and sardine cooking wastewaters.

In this work, the membrane was used to permeate the target peptides. For the purification of bioactive peptides with certain MW, the selection of the MWCO of the membranes was made according to the characterization of the raw materials by FPLC, as shown in Figure 1. After the selection of the membranes, the membrane experiments were performed in the dead-end mode under controlled transmembrane pressure using flat sheet membranes at 0.1 and 1.0 bar with one module (Amicom, Merck, Darmstadt, Germany, 45.4 × 10^−4^ m^2^ membrane area) and at 5.0 bar with another module (METCell, Membrane Extraction Technology, London, UK, 51.4 × 10^−4^ m^2^ membrane area). The permeate mass was measured with the an electronic balance (Kern 572, Kern, Frankfurt, Germany). The feed vessel was stirred at 500 rpm, processing 200 g of codfish blood and sardine cooking wastewaters. The scheme of the ultrafiltration experiment is shown in Figure 2.

The permeability *Lp* [L/(m^2^ h bar)] was calculated through Equation (1):

(1) $$L_{p=\frac{V_{perm}}{A,t.TMP}}$$

where *V_perm_* [L] is the permeate volume, *t* [h] is the time of permeation, *A* [m^2^] is the membrane area, and *TMP* [bar] is the transmembrane pressure.

The observed rejection of the protein/peptides for each membrane under study, at the end of each experiment (at 80% permeate recovery, equivalent to the concentration of the retentate 5×), was calculated through Equation (2):

(2) $$R[\%]=1-\frac{C_{peptides,\;PFG}}{C_{peptides,FF}}$$

where *c_peptides,PFG_* and *c_peptides,FF_* are, respectively, the concentrations of the protein/peptides in the permeate (the total accumulated permeate) PFG, and in the retentate FF at the end of each experiment.

In Equation (2), the protein/peptides concentrations of each sample obtained were calculated by the product of their calibration factor and their chromatograms areas measured for each sample *A_peptides_*. For FPLC analyses, considering that the calibration factor of the protein/peptides is constant in the all samples analyzed, Equation (2) was converted in Equation (2′):

(2′) $$R[\%]=1-\frac{A_{peptides,\;PFG}}{A_{peptides,FF}}$$

where *A_peptides,PFG_* and *A_peptides,FF_* are, respectively, the chromatogram areas obtained by the FPLC in the accumulated permeate and in the retentate at the end of each experiment.

Additionally, in order to access the quality of the analytical data in each membrane experiment, partial mass balances to the protein/peptides were calculated through Equation (3), which was converted in Equation (3′) for the FPLC analysis (in a similar conversion of Equation (2) to Equation (2′):

(3) $$c_{peptides,IF}{\times m}_{IF}{=c}_{peptides,FF}{\times m}_{FF}{+c}_{peptides,PFG}{\times m}_{PFG}$$

(3′) $$1=\frac{A_{peptides,FF}{\times m}_{FF}}{A_{peptides,IF}{\times m}_{IF}}+\frac{A_{peptides,PFG}{\times m}_{PFG}}{A_{peptides,IF}{\times m}_{IF}}$$

where *A_peptides,IF_* is the chromatogram area obtained by the FPLC measurements and where the *m_IF_*, *m_FF_*, and *m_PFG_* are, respectively, the total mass of the initial feed (pre-treated raw materials), and retentate and permeate at the end of each experiment.

### 2.3. Analytical Methods

Pre-treated codfish blood and sardine cooking wastewaters and the corresponding retentates and permeates, at the end of each membrane filtration experiment, were chemically characterized by Kjeldahl and by FPLC. The pre-treated raw materials and the corresponding permeates were also characterized in terms of the biological properties of antioxidant activity (by ABTS^+^ and ORAC), antimicrobial activity (growth inhibition curves), and antihypertensive activity (by ACE inhibitory fluorimetric assay).

#### 2.3.1. Measurement of the Content in Protein and Peptides

The protein content of selected samples (see Section 2.3) was determined in duplicates by Kjeldahl [41] and then used for the calculation of the total protein content by multiplying the conversion factor of 6.38. The molecular weight distribution of the selected samples was also determined in duplicates by gel filtration chromatography using the FPLC AKTA pure 25 system (GE Healthcare Life Sciences, Uppsala, Sweden), which consisted of two gel filtration columns—the Superdex^®^ 200 10/300 GL and Superdex Peptide 10/300 GL. The eluent used was 0.05 M phosphate buffer (pH 7.0), with 0.15 M NaCl and 0.2 g/L NaN_3_ at a 0.5 ml/min flow rate. Eluent absorption was tracked at 280 nm and thyroglobulin (669 kDa); aldolase (158 kDa); conalbumin (75 kDa); ovalbumin (43 kDa); carbonic anhydrase (29 kDa); ribonuclease A (13.7 kDa) from Sigma-Aldrich, St. Louis, MI, USA and whey peptide (1.2 kDa) (KGYGGVSLPEW, GeneScript Piscataway, NY, USA), were used to calibrate the system. Each protein/peptide quantification was assessed by the k integration of the peak areas.

#### 2.3.2. Measurement of Antioxidant Activity

The measurement of the antioxidant capacity of the different samples (see Section 2.3) were carried out in triplicates by the methods ABTS^+^ radical scavenging activity (as in Re et al. [42]) and ORAC. In brief, ABTS^+^ radical cation was formed from the reaction of 7 mM 2,20-azinobis (3-ethylbenzothiazoline-6-sulfonic acid) diammonium salt (ABTS^+^) and 2.45 mM potassium persulfate (Sigma–Aldrich both, St. Louis, MO, USA) after room temperature incubation for 16 h in the dark. The ABTS^+^ solution was mixed with distilled water to an absorbance of 0.70 ± 0.02 at 734 nm. After the addition of 1.0 mL ABTS^+^ solution to 10 μL of sample, the absorbance of the mixture was performed upon 6 min. The inhibition percent of the sample was compared to that of a standard curve drown using ascorbic acid. The oxygen radical absorbance capacity (ORAC) test was based on the one defined by Contreras et al. [43]. In brief, the reaction was conducted at 40 °C in 75 mM of phosphate buffer (pH 7.4) and the final assay mixture (200 μL) of fluorescein (70 nM), AAPH (14 mM), and either antioxidant (Trolox (9.98 × 10^−4^–7.99 × 10^−3^ μmol/mL) or sample (the feed in each membrane filtration experiment and the corresponding permeates). The fluorescence has been reported for 137 minutes (104 cycles). A FLUOstar OPTIMA plate reader (BMG Labtech, Offenburg, Germany) with 485 nm excitation and 520 nm emission filters was used. The system was run by the FLUOstar Control software version (1.32 R2) for fluorescence evaluation. Final ORAC values were reported as μmol Trolox equivalent (TE)/mg sample.

#### 2.3.3. Evaluating of Antimicrobial Activity

The different samples (see Section 2.3) were screened for antimicrobial activity against a Gram-negative bacteria *Escherichia coli* (ATCC 25922), a Gram-positive bacteria methicillin-sensitive *Staphylococcus aureus* (MSSA) (ATCC 25923), and a yeast *Candida albicans* (CCGU 49242). Extract solutions at 4% were prepared using Mueller Hinton Broth (MHB) (Biokar Diagnostics, Beauvais, France) and inoculated (V/V) using an overnight inoculum (ca. 10^-5^ CFU/ mL). Two-hundred and fifty μL of each mixtures was transferred to a 96-well microtiter (Nunc, Darmstadt, Germany) and the optical density (OD) was measured at 660 nm for a 24 h duration at 37°C (1 h intervals) using a microplate reader (Fluostar, Optima; BMG Labtech, Ortenberg, Germany), the increase in OD was considered to be the result of bacterial growth. Positive control was drawn using inoculated *Escherichia coli*, *Staphylococcus aureus*, and *Candida albicans* without an antimicrobial agent and sterile MHB was used as a negative control. Each condition has been evaluated as a triplicate.

#### 2.3.4. Measurement of Antihypertensive Activity

The ACE-inhibitory effect of the different samples (see Section 2.3) was assessed in triplicate using the fluorimetric assay of Sentandreu and Toldrá [44] as updated by Quirós, Contreras, Ramos, Amigo, and Reci [45]. The ACE (peptidyl-dipeptidase A, EC 3.4.15.1) has been ordered from Sigma Chemical (St. Louis, MO, USA). The ACE working solution was mixed with 0.15 M Tris buffer (pH 8.3) comprising 0.1 mM ZnCl_2_ and 0.04 U/mL of enzyme in the final reaction solution. A total of 40 mL of distilled water or working solution was applied to each microtiter-plate well and then modified to 80 mL by applying distilled water to blank (B), control (C) or samples (S). The enzyme reaction was started by adding 160 mL of 0.45 mM o-Abz-Gly-p-Phe(NO_2_)-Pro-OH (Bachem Feinchemikalien, Bubendorf, Switzerland) dissolved in 150 mM Tris-based buffer (pH 8.3), comprising 1.125 M NaCl, and incubated at 37 °C. The fluorescence generated was assessed at 30 min with a FLUOstar OPTIMA plate reader (BMG Labtech, Offenburg, Germany). Ninety-six-well microplates (Porvair, Leatherhead, UK) were used. Emission and excitation wavelengths were 420 and 350 nm, respectively. The software used to process data was FLUOstar control (version 1.32 R2, BMG Labtech). The activity of each sample was analyzed in duplicate. The inhibiting activity was defined as the concentration of peptide required to inhibit the original activity by 50% (IC50). The ACE-inhibitor activity level was calculated through Equation (4):

(4) 100×(C−S)/(C−B)

Non-linear fitting of data was performed to calculate the IC50 values, as previously done by Quirós et al. [46]. For this assay, the protein content of the peptide fractions was determined by the Kjeldahl method.

## 3. Results and Discussion

### 3.1. Characterization of the Raw Materials in Terms of Their Content in Protein/Peptides and Membrane Selection

The pre-treated raw materials codfish blood and sardine cooking wastewaters (see the pre-treatment carried out in Section 2.1) were characterized in terms of their content in protein/peptides by Kjeldahl method, respectively 0.44 ± 0.02 g/mL and 0.30 ± 0.01 g/mL.

The samples (pre-treated codfish blood and sardine cooking wastewaters) were also evaluated by size exclusion FPLC. The chromatogram areas for the different range of MWs of different protein/peptides (> 10 kDa and < 1 kDa) are reported in Table 3.

The FPLC data showed that both pre-treated raw materials exhibited a similar pattern in terms of the size distribution of their peptides, related to their chromatogram area distribution with two distinct groups of peptides (as the FPLC data of untreated raw materials, data not shown): the group of peptides with the highest molecular weights MWs/sizes (>10 kDa) and the group of peptides with the lowest MWs (<1 kDa). The percentage of peptides in the group with the lowest MWs represented around 80% of the total protein/peptides of each raw material; therefore., the samples had mostly small peptides. Nevertheless, this could be explained by the fact that both raw materials had similar storage conditions before being processed, which might have led to this similar hydrolysis pattern.

Considering the FPLC characterization, pressure-driven membrane technologies, using a membrane with an appropriate MWCO, may permeate the target compounds with a certain MW [47]. Codfish blood was decided to be processed with membranes with different MWCO (≥10 kDa MWCO, in the range of the values of MWs of the group of peptides with the highest MWs) combined with different membrane material—PW and MW from GE Suez and UP010 from Microdyn Nadir. A low, mild value of transmembrane pressure of 0.1 bar was chosen, not to promote membrane fouling and due to the relatively high MWCO values of the selected membranes. Sardine cooking wastewaters were decided to be processed with a membrane with a lower MWCO (equal to 2.5 kDa, an MWCO slightly above the values of MWs of the group with peptides with the lowest MWs): GH from GE Suez operated with different transmembrane pressures (1.0 and 5.0 bar), in a membrane with the highest stability at high-pressure values of the membranes under study (with a maximum operating pressure of 27.57 bar). The characteristics of the selected membranes are summarized in Section 2.1, Table 2.

### 3.2. Fractionation of the Raw Materials: Protein/Peptides Characterization of the Fractions and Evaluation of Membrane Performance

The objective of using membrane technology is the fractionation and purification of small bioactive peptides from fish by-products (codfish blood and sardine cooking wastewaters). The effect of the membrane pore size (related to MWCO), combined with membrane material (when processing codfish blood, see Section 3.1) and the effect of the transmembrane pressure (when processing sardine cooking wastewaters, see Section 3.1) on the performance of the membrane were studied in terms of the observed rejection of the target peptides (desirably low) and of the permeabilities (desirably high), at room temperature.

Codfish blood and sardine cooking wastewaters (the initial feed in each membrane filtration experiment) and the corresponding retentates and permeates at the end of each membrane filtration experiment were characterized in terms of their total protein/peptides content by Kjeldahl method and by different MWs using size-exclusion FPLC. This allowed relating the concentration and sizes of the peptides of each sample with their biological properties.

#### 3.2.1. Codfish Blood

**Figure 3:**
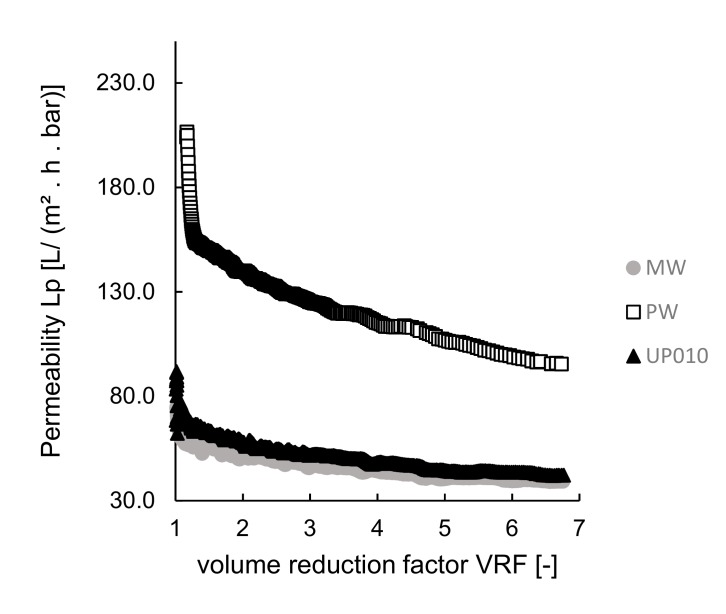
Ultrafiltration experiments at room temperature using various membranes: permeabilities plotted as a function of the volume reduction factor, VRF.

The performance of the membranes was assessed by measuring permeabilities. The permeabilities of the different membranes were plotted against the volume reduction factor, VRF [–] (the ratio between the initial feed volume and the retentate volume), as presented in Figure 3. When comparing PW and UP010 membranes with the same MWCO (10 kDa), the different values of permeability obtained could be explained by the hydrophilic behavior of the membranes, measured by the contact angles, which were much different (see Table 2). In fact, membrane PW had a lower contact angle (22.86° ± 1.36°), and thus, a higher hydrophilic behavior, than membrane UP010 (with a contact angle of 56.30° ± 2.46°), which could explain the higher permeability values when PW membrane is used. As both PW and UP010 commercial membranes are comprised of the same polymer material (polyethersulfone), the higher hydrophilicity behavior of PW might be explained by a treatment performed by the producer to turn the membrane more hydrophilic.

When comparing MW and UP010 membranes (with similar hydrophilic behavior, related to similar contact angles), the permeabilities when using MW membrane were expected to be higher, due to its higher MWCO/larger pore sizes (MWCO of 20—50 kDa or 50 kDa, see Table 2) than UP010 10 kDa membrane). Considering that codfish blood is a naturally complex matrix, most probably in the presence of small compounds, which may cause membrane fouling, a membrane with larger pore size is more likely to have intrapore fouling, which contributes to a decrease of fluxes and permeabilities, similarly to what was reported for microalgae processing [48].

The protein and peptides characterization of the retentates and permeates (at the end of each membrane filtration experiment) of the pre-treated codfish blood was achieved by the Kjeldahl method, as presented in Table 4, and by FPLC, as shown in Table 5.

The FPLC analysis showed that for all membranes tested, the group of peptides with the highest MWs (>10 kDa) did not permeate (corresponded to an observed rejection of the larger size peptides of 100%), as aimed. Nevertheless, the permeation of the group of peptides with the lowest MWs (<1 kDa), still has to be evaluated.

Table 6 shows the mass balance closure in relation to total protein/peptides by both Kjeldahl and FPLC methods, the observed rejection of the total protein/peptides by Kjeldahl method and the observed rejection of the group of peptides with the lowest MWs (<1 kDa) by FPLC when processing codfish blood.

Regarding the Kjeldahl results, all the mass balances closed within less than Ca. 10%, assessing the good quality of the analytical data. Regarding the FPLC results, when processing with the MW and UP010 membranes, the mass balances closed within less than around 13%, which was still reasonable. The rejection of the peptides given by the Kjeldahl method was higher than the rejection of the peptides given by the FPLC method, as expected because the latter only took into account small peptides (<1 kDa), which preferentially permeates. Therefore, the rejection of the peptides given by the Kjeldahl method was not so adequate to be analyzed as it combined the two groups of peptides mentioned above (see Section 3.1), with a very different permeation behavior.

When processing with PW, the permeation behaviors were very different, although it corresponded to the highest values of permeability. Regarding FPLC analyses, the mass balances closed within Ca. 20%, probably because some of the small peptides (1 kDa) were adsorbed on the membrane, together with the larger protein/peptides, and were rejected. That was in line with its highest rejection of small peptides of the three membranes tested and with the fact that the rejection of total peptides given by the Kjeldahl method was lower than that of the small peptides (<1kDa) given by the FPLC method.

Comparing MW and UP010 membranes, with similar hydrophilic behavior, the rejection of small peptides when using MW was higher, despite its higher MWCO, which is in line with a higher fouling phenomenon.

The best performing membrane was UP010, with a minimum rejection of 2% of the small peptides, although the permeability was not as good as when the PW membrane was used.

#### 3.2.2. Sardine Cooking Wastewaters

**Figure 4:**
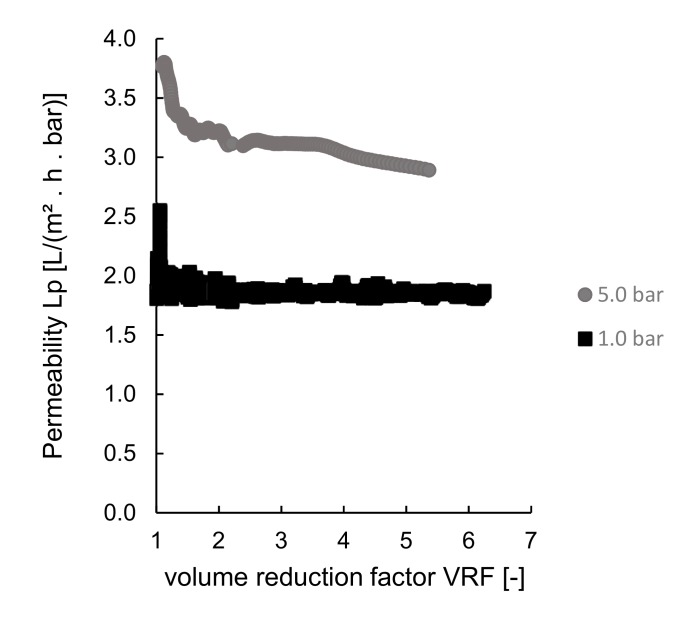
Ultrafiltration experiments at room temperature using different pressures: permeability plotted as a function of the volume reduction factor, VRF.

Pre-treated sardine cooking wastewaters were processed with the GH membrane from GE Suez at 1.0 bar and 5.0 bar transmembrane pressures. The permeabilities at different transmembrane pressure are plotted against the adimensional volume reduction factor, VRF [–], as presented in Figure 4.

As expected, the increase of transmembrane pressure (the driving force of the process) led to an increase in permeabilities (Figure 4).

The protein/peptides characterization of the retentates and permeates (at the end of each membrane filtration experiment) of the pre-treated sardine cooking wastewaters was achieved by the Kjeldahl method, as presented in Table 7, and by FPLC, as shown in Table 8.

As in the previous section, the FPLC data shows that, at 1.0 and 5.0 bar, the group of peptides with the highest molecular weights MWs/sizes (>10 kDa) did not permeate (corresponded to an observed rejection of the larger size peptides of 100%), as aimed.

Table 9 shows the mass balance closure in relation to total protein/peptides by both Kjeldahl and the FPLC methods, the observed rejection of the total protein/peptides by Kjeldahl method and the observed rejection of the group of peptides with the lowest molecular weights MWs/sizes (<1 kDa) by the FPLC, when processing sardine cooking wastewaters.

All the mass balances closed within less than around 4% for Kjeldahl analysis and closed within less than around 11% for FPLC analysis, which is reasonable (better for the Kjeldahl analyses, as on the previous section).

At 5.0 bar, and although it corresponded to the highest values of permeability, the rejection of total peptides given by the Kjeldahl method was lower than the rejection of the small peptides (< 1 kDa) given by the FPLC method, which is probably related to the fact that small peptides are adsorbed on the membrane, causing intrapore fouling. This probable fouling effect is typical when the transmembrane pressure increases, and it is in line with a higher rejection of small peptides (< 1kDa) given by FPLC.

The experiment with the best results was at 1.0 bar, with a minimum rejection of 23% of the small peptides, although the permeability was not as good as at 5.0 bar.

### 3.3. Biological Properties of the Raw Materials and Corresponding Permeates from Ultrafiltration

The aim of producing fractions through UF processing, in which the target fraction is the permeate, is to: (1) obtain permeates with a maximized concentration of peptides (corresponding to low values of observed rejections of peptides); (2) eliminate large compound contaminants with higher molecular mass, which may reduce their biological properties (3) obtain permeates with better global antioxidant, antimicrobial and antihypertensive activities, and hence, better bioactivity properties than the non-fractionated raw materials. For codfish blood processing, the membrane experiments were performed at 0.1 bar, a low value that enhances low observed rejections of target peptides, at room temperature. Sardine cooking wastewaters were processed with the GH membrane from GE Suez at 1.0 bar and 5.0 bar values of transmembrane pressure. Codfish blood and sardine cooking wastewaters (the feed in each membrane filtration experiment) and the corresponding permeates, at the end of each membrane filtration experiment, were evaluated for their biological properties of antioxidant activity by (ABTS^+^ and ORAC), antimicrobial activity, and antihypertensive activity by the fluorimetric assay. This allows evaluating the biological properties achieved by membrane processing, assessing whether peptide fractions exhibit improved biological properties than raw materials, turning these fractions into potential high-value functional ingredients in food formulations or cosmetic/pharmaceutical products.

#### 3.3.1. Antioxidant Activity of Purified Peptides

The most appealing properties of antioxidant peptides are their capacities, such as metal ion chelating activity and free radical and ROS scavenging activity, to exhibit few side effects in humans due to their natural sources [49].

The exact mechanism of action of antioxidant peptides has not been completely established. Wang et al., [50] indicated that tyrosine, tryptophan, methionine, lysine, cysteine, proline, and histidine in the sequence improved the antioxidant activity of antioxidant peptides through proton-donating and hydrogen atom exchange capability. The negatively charged acidic Glutamic acid causes free radical scavenging activity due to the presence of excess electrons [49]. The research revealed that peptides or protein hydrolysates with a high content of aromatic amino acid demonstrated high free radical scavenging activity [51,52].

**Figure 5:**
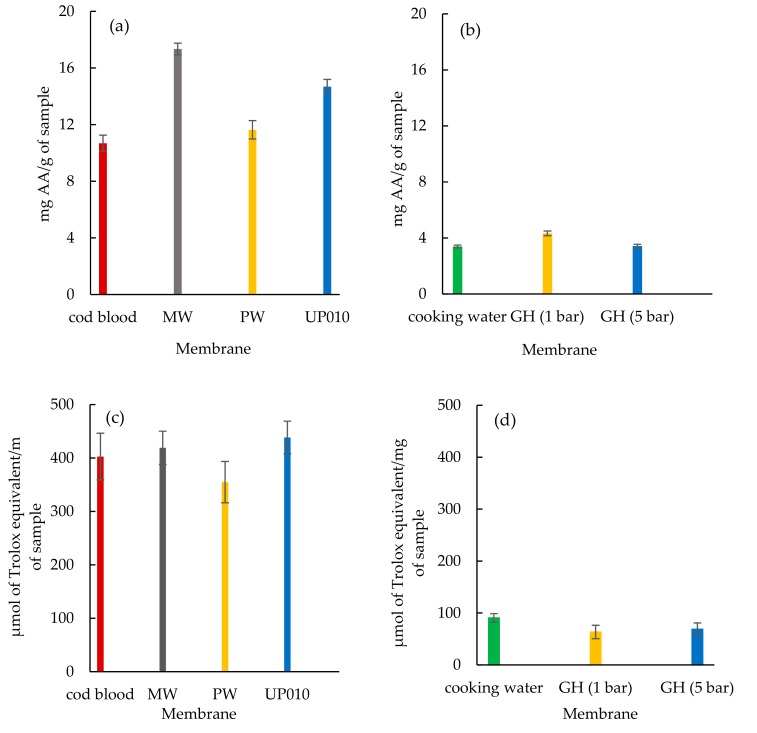
Antioxidant activities of fish by-products (codfish blood and sardine cooking wastewaters) and corresponding peptide fractions. (**a**) ABTS^+^ radical scavenging activity for codfish blood and the corresponding peptide fractions from (MW, PW, and UP010 membranes), (**b**) ABTS^+^ radical scavenging activity for cooking wastewaters and the corresponding peptide fractions from the GH membrane, using 1.0 bar and 5.0 bar of transmembrane pressure, (**c**) ORAC value for codfish blood and the corresponding peptide fractions from (MW, PW, and UP010 membranes) and (**d**) ORAC value for sardine cooking wastewaters and the corresponding peptide fraction from the GH membrane using 1.0 bar and 5.0 bar of transmembrane pressure. The results are shown as mean ± S.D. with three determinations.

To verify the antioxidant activity of the purified peptides, fish by-products (codfish blood and sardine cooking wastewater) and the corresponding permeates were subjected to investigation of their ABTS^+^ radical scavenging activity and ORAC. As shown in (Figure 5a,b), the peptide fractions showed varying ABTS^+^ radical scavenging activity per g of sample. The peptide fractions from MW and UP010 membranes illustrated the highest ABTS^+^ radical scavenging activity of 17.34 ± 0.41 and 14.68 ± 0.50 mg AA ascorbic acid/g, respectively, while codfish blood and the peptide fractions from the PW membrane showed weaker ABTS^+^ radical scavenging activity of 10.68 ± 0.57 and 11.63 ± 0.65 mg AA/g, respectively. The peptide fractions from UP010 and MW membranes showed higher ORAC values of 438.37 ± 30.54 and 419.10 ± 31.09 µmol of Trolox equivalent/mg, respectively, in comparison with codfish blood and the peptide fractions from PW membrane of 402.67 ± 43.62 and 354.87 ± 38.61 µmol of Trolox equivalent/mg, respectively. (Figure 5c). The ABTS^+^ radical scavenging activity results demonstrated a slight improvement in the antioxidant activity of peptide fractions from the GH membrane using 1.0 bar of transmembrane pressure compared to sardine cooking wastewaters (Figure 5b). On the contrary, the improvement of ABTS^+^ radical scavenging activity in peptides fraction from the GH membrane (1.0 bar), the ORAC value in both peptide fractions obtained from the GH membrane decreased and showed reduced power than sardine cooking wastewaters (Figure 5d).

Overall, it appeared that both peptide fractions from UP010 and MW membranes effectively increased ABTS^+^ radical scavenging activity and ORAC than peptide fractions from the PW membrane and codfish blood. The peptide fraction from the GH membrane when using 1.0 bar of the pressure showed a better ABTS^+^ radical scavenging activity compared to sardine cooking wastewaters and peptide fractions from the GH membrane when using 5.0 of the pressure.

#### 3.3.2. Antimicrobial Activity of Purified Peptides

**Figure 6:**
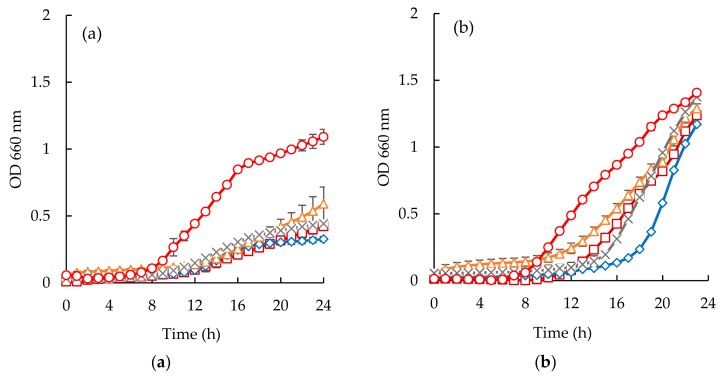

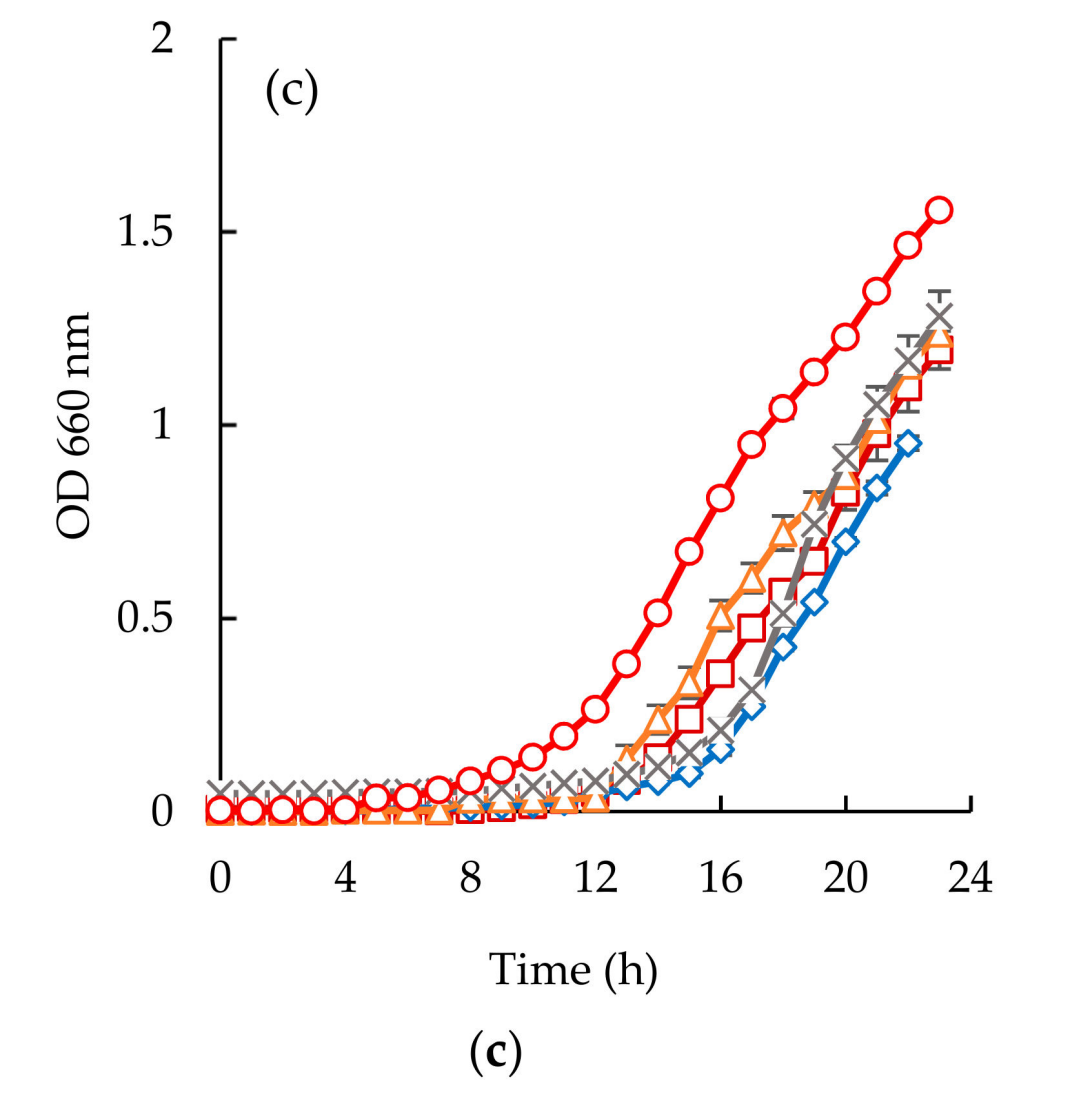
Time inhibition curves drawn at different permeates obtained from codfish blood (○, positive control; □, codfish blood; x, GE Suez MW; Δ, GE Suez PW; ◊, Microdyn Nadir UP010 for *E. coli* (**a**), *S. aureus* (**b**), *C. albicans* (**c**).

**Figure 7:**
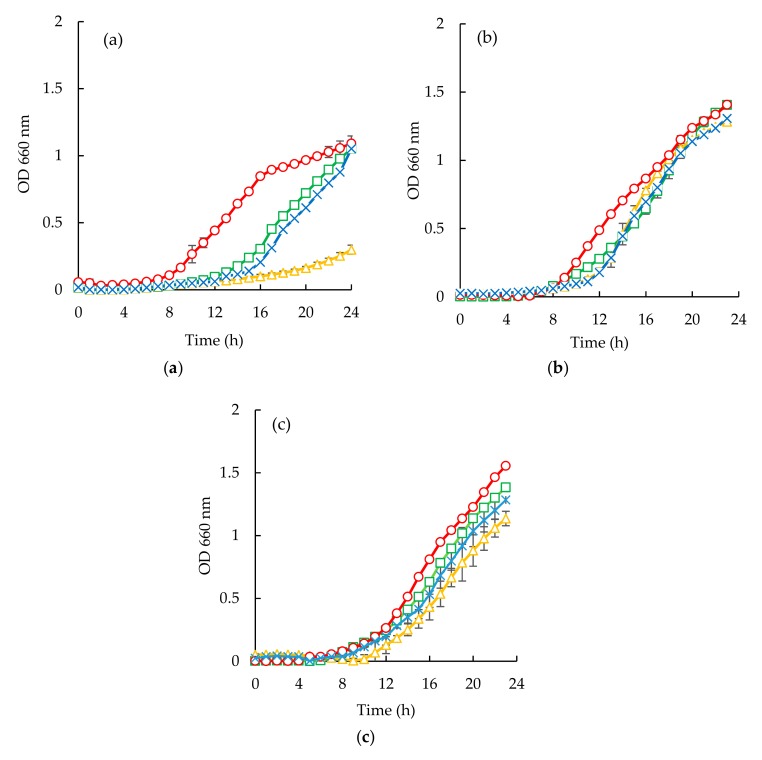
Time inhibition curves drawn with permeates obtained from sardine cooking wastewaters (○, positive control; □, cooking wastewater; Δ, GE Suez GH (1.0 bar); x, GE Suez GH (5.0 bar) for *E. coli* (a), *S. aureus* (b), *C. albicans* (c).

*Escherichia coli* appeared to be the most susceptible microorganism to the action of the purified peptide (Figure 6a and Figure 7a). From the analysis of (Figure 6a and Figure 7a), it can be seen that a concentration of 40 mg/mL was capable of reducing microbial growth. In fact, sardine cooking wastewaters, codfish blood, and the corresponding permeates caused an increase of lag phase duration, and cause reduction of bacterial growth at the 24 h mark, for example, peptides fraction from the GH membrane when using 1.0 bar of the pressure was capable of delaying the beginning of exponential growth by 3 h.

Considering the *Staphylococcus aureus* strain, it is interesting to highlight the different behaviors observed between the two different samples (Figure 6b and Figure 7b). Codfish blood and obtained permeates caused a significant delay of the lag phase, while sardine cooking wastewaters and corresponding permeates had no effect on the OD after incubation for 24 h. Besides, the strain of *Staphylococcus aureus* exhibited higher lag phases when exposed to permeates of UP010 and MW compared to codfish blood, and the PW permeate. It is interesting to note that, peptide fractions from UP010 membrane appeared to induce the highest reduction of OD, while the other permeates exhibited an OD, after 24 h, similar to the one observed for the positive control although the maximum OD registered over the 24 h period was lower than the one registered for the positive control (Figure 6b).

For *Candida albicans* (Figure 6c and Figure 7c), it can be seen that codfish blood and the corresponding permeates caused a significant delay of the lag phase after 24 h. The growth curves for the sardine cooking wastewaters and the corresponding permeates exhibited no differences to the positive control.

#### 3.3.3. ACE inhibitory Activity of Purified Peptides

Numerous studies have shown that dietary protein consumption can lead to the reduction of high blood pressure, coronary heart disease, and other infarctions risk [53]. Some natural ACE inhibitor products have now been established as dietary supplements that promote healthy blood pressure, including Bonito peptide, Vasotensin^R^, and PeptAce™ peptides isolated from fish bonito (*Sarda orientalis*) [54]. These products do not seem to have any side effects in clinical studies [54], maybe because of the specific mechanism of action in inhibiting ACE between fish peptides and drugs. Antihypertensive activity is based on inhibition of a crucial regulator of blood pressure, angiotensin-converting enzyme (ACE), which could degrade bradykinin vasodilator molecule and thereby decrease hypertension through ACE inhibitors. In some work with fish protein hydrolysates/peptides, antihypertensive peptides were extracted [20,23].

To better understand the role of membrane processing, further characterization of ACE inhibition by codfish blood, sardine cooking wastewaters, and the corresponding permeates at the end of each membrane filtration experiment, IC50 was determined. As shown in Table 10, codfish blood and peptide fractions from MW, PW, and UP010 membranes showed no inhibition (IC50 > 2000 µg protein/mL) as well as sardine cooking wastewaters and peptide fractions from the GH membrane (IC50 > 1000 µg protein/mL).

In the present work, for all the samples, IC50 concentration was significantly high, which indicates that none of the samples had an activity that can be considered antihypertensive.

## 4. Conclusions

This work proposes the sustainable production of fish by-products (codfish blood and sardine cooking wastewaters) fractions enriched in small biopeptides, free from large compound contaminants, with enhanced biological properties than the raw materials at laboratory scale. This process uses membrane technologies (ultrafiltration) that allow obtaining permeate fractions enriched in small bioactive peptides. The Kjeldahl method, combined with the FPLC characterization of fish by-products and the corresponding permeate fractions, allowed an appropriate design of the overall process and definition of the most interesting fractions to be targeted for functional properties. Results of antioxidant activity suggest that codfish blood is a potential source of natural antioxidant peptides. Therefore, these purified peptides may possibly be used as a functional food additive for the reduction of oxidative stress-associated diseases and the inhibition of oxidation in foods. Furthermore, the purified peptides from codfish blood and from sardine cooking wastewaters also exhibited an interesting antimicrobial activity against *Escherichia coli*, which appears as the most susceptible microorganism. The antimicrobial activity results bring some insight into the potential of the fish by-products biopeptides, strengthening the importance of additional studies to further exploit the purified peptides potential as new, alternative antimicrobial agents. The best results in terms of low rejection of the target compounds (2%) and in terms of good antioxidant activity (14.68 ± 0.50 mg Ascorbic Acid/g and 438.37 ± 30.54 µmol of Trolox equivalent/mg) and good antimicrobial properties against *E. coli*, corresponded to the permeate of membrane UP010 when codfish blood was processed. As future work, this study will be conducted at the pilot scale where membrane cleaning procedures will be defined to the selected membrane.

## Figures and Tables

**Table 1:** Biopeptides from fish by-products with antioxidant, antimicrobial, and ACE inhibitory activities.

Fish by-Products	Molecular Weight (Da)	Bioactive Potential	References
Tuna backbone protein	1519.00	Antioxidant	[8]
Alaska pollack (*Theragra chalcogramma*) frame protein	672.00	Antioxidant	[9]
Seabass (*Lates calcarifer*) skin	364.00	Antioxidant	[28]
Bluefin leatherjacket (*Navodon septentrionalis*) skin	389.41	Antioxidant	[29]
	546.63		
	432.52		
Unicorn leatherjacket (*Aluterus monoceros*) skin	555.27	Antioxidant	[30]
	511.29		
	557.30		
	472.24		
Tuna (*Thunnus obesus*) dark muscle	1222.00	Antioxidant	[31]
Anchovy (*Setipinna taty*) half-fin	1100.00 to 1700.00	Antimicrobial	[32]
Atlantic mackerel (Scomber scombrus) coproducts	<1200.00	Antimicrobial	[33]
Yellow catfish (*Pelteobagrus fulvidraco*) skin mucus	2244.40	Antimicrobial	[34]
Skate (*Raja Kenojei*) skin	975.38	ACE inhibitory	[15]
	874.45		
Bluefin tuna (*Thunnus thynnus*) frame	2482.00	ACE inhibitory	[17]
Atlantic salmon (*Salmo salar L.*) skin	<1000.00	ACE inhibitory	[20]
Skate (*Okamejei kenojei*) skin gelatin	829.00	ACE inhibitory	[23]
	720.00		
Pangasius catfish (*Pangasius sutchi*) skin and bone gelatins	<1000.00	ACE inhibitory	[24]

**Table 2:** Properties of the membranes analyzed (according to the manufacturers’ data).

Membrane	Membrane Material [39,40]	Retention (kDa) [39,40]	Maximum Operating Pressure (bar) [39,40]	Maximum Operating Temperature (°C) [39,40]	Contact Anglea (°)
MW, GE Suez	Hydrophilic PAN	50 (on proteins); ~20–50 (extremely hydrophilic)	6.9	50	62.55 ± 3.74
PW, GE Suez	PES	10 (for dextran) (>96% rejection of Cytochrome-C (13,300 MW proModel1 A B2 C3 Weight lbs (kg)	13.8	50	22.86 ± 1.36
UP010, Microdyn Nadir	PES	10	10.0	55	56.30 ± 2.46
GH, GE Suez	PA-TFC	2.5 (on polyethylene glycol)	27.6	50	59.17 ± 1.31

Legend: MWCO molecular weight cut-off, PAN Polyacrylonitrile, PES Polyethersulfone, PA Polyamide, TFC thin-film composite. ^a^ Contact angles of the membranes with the water were measured by the sessile drop method in a contact angle goniometer (Attension Theta Flex, Sweden).

**Table 3:** FPLC chromatogram area of the different peaks from the pre-treated codfish blood and sardine cooking wastewaters (raw materials). The results are given in (mL*mAU).

Molecular Weight (kDa)	Codfish Blood	Sardine Cooking Wastewaters
	Area (mL*mAU)	Area (mL*mAU)
≥10	79.19 ± 0.69	27.50 ± 1.33
≤1	382.59 ± 2.21	261.05 ± 0.77

**Table 4:** Composition of the retentates and permeates of the pre-treated codfish in terms of their content in proteins/peptides by Kjeldahl method. Results are given in g per 100 mL of sample.

Membrane	Sample	Total Protein [g/100 mL]
GE Suez MW	Retentate (FF)	0.75 ± 0.05
	Total accumulated permeate (GFP)	0.31 ± 0.00
GE Suez PW	Retentate (FF)	0.62 ± 0.02
	Total accumulated permeate (GFP)	0.44 ± 0.00
Microdyn Nadir UP010	Retentate (FF)	0.73 ± 0.04
	Total accumulated permeate (GFP)	0.37 ± 0.01

Legend: FF Final feed (retentate), GFP Global final permeate.

**Table 5:** FPLC chromatogram areas of the peaks from the retentates and permeates of pre-treated codfish obtained from different membranes. Results are given in (mL*mAU).

Molecular weight (kDa)	Membrane					
	GE Suez MW		GE Suez PW		Microdyn Nadir UP010	
	FF	GFP	FF	GFP	FF	GFP
	Area (mL*mAU)	Area (mL*mAU)	Area (mL*mAU)	Area (mL*mAU)	Area (mL*mAU)	Area (mL*mAU)
≥ 10	204.03 ± 7.27	—	205.48 ± 8.07	—	257.77 ± 4.70	—
≤ 1	477.46 ± 16.78	299.27 ± 12.9	400.26 ± 1.24	308.63 ± 1.60	403.75 ± 7.32	396.21 ± 4.03

Legend: FF Final feed (retentate), GFP Global final permeate.

**Table 6:** Membrane processing of codfish blood: mass balance of total protein/peptides by Kjeldahl and the FPLC methods, the observed rejection of total protein/peptides by Kjeldahl method, and the observed rejection of peptides (with MWs < 1 kDa) by the FPLC method.

Membrane	Kjeldahl		FPLC	
	Mass Balance Closure [%]	R [%]	Mass Balance Closure [%]	R (<1kDa) [%]
GE Suez MW	90±6	59±3	87±4	23±0
GE Suez PW	103±3	30±1	81±5	37±2
Microdyn Nadir UP010	101±6	48±5	94±2	2±0

**Table 7:** Composition of protein/peptides of the peaks of the retentates and permeates of the pre-treated sardine cooking wastewaters obtained when using the GH membrane at 1.0 bar and 5.0 bar transmembrane pressures by Kjeldahl method. The results are given in g per 100 mL of sample.

Transmembrane Pressure (bar)	Sample	Total Protein [g/100mL]
1.0	Retentate (FF)	0.38 ± 0.02
	Total accumulated permeate (GFP)	0.22 ± 0.02
5.0	Retentate (FF)	0.57 ± 0.01
	Total accumulated permeate (GFP)	0.22 ± 0.01

Legend: FF Final feed (retentate), GFP Global final permeate.

**Table 8:** FPLC chromatogram areas of the peaks from the retentates and permeates of pre-treated sardine cooking wastewaters when using the GH membrane at 1.0 bar and 5.0 bar transmembrane pressures.

Molecular Weight (kDa)	Transmembrane Pressure [bar]			
	1.0		5.0	
	FF	GFP	FF	GFP
	Area (mL*mAU)	Area (mL*mAU)	Area (mL*mAU)	Area (mL*mAU)
≥ 10	231.15 ± 2.74	—	36.27 ± 20.93	—
≤ 1	575.32 ± 4.03	171.78 ± 1.03	311.66 ± 10.17	239.09 ± 7.23

Legend: FF Final feed (retentate), GFP Global final permeate.

**Table 9:** Membrane processing of sardine cooking wastewaters: mass balance of total protein/peptides by Kjeldahl and FPLC methods, the observed rejection of total protein/peptides by the Kjeldahl method, and the observed rejection of peptides (with MW < 1 kDa) by FPLC method.

Transmembrane Pressure (bar)	Kjeldahl		FPLC	
	Mass Balance Closure [%]	R [%]	Mass Balance Closure [%]	R (1< kDa) [%]
1.0	99 ± 9	41 ± 3	89 ± 5	23 ± 1
5.0	96 ± 6	61 ± 4	100 ± 1	70 ± 1

**Table 10:** Results of ACE inhibitory of codfish blood, sardine cooking wastewaters, and the corresponding permeates.

Sample	ACE-inhibitory Activity (IC50, µg/mL)
Codfish blood	>2000
Permeate (GE Suez MW)	>2000
Permeate (GE Suez PW)	1323 ± 131
Permeate (Microdyn Nadir UP010)	2019 ± 228
Sardine cooking wastewaters	1332 ± 94
Permeate (GE Suez GH) (1.0 bar)	>2000
Permeate (GE Suez GH) (5.0 bar)	>2000

Legend: Data are expressed as mean values ± SD of two experiments. IC50: Concentration needed to inhibit angiotensin-converting enzyme activity by 50%.
